# Students’ participation in Hult Prize and their decision for entrepreneurship: Data gathered from Hult Prize 2018 regional finals in Nigeria

**DOI:** 10.1016/j.dib.2018.05.089

**Published:** 2018-05-23

**Authors:** Stephen Oluwatobi, Damilare Oshokoya, Aderemi Atayero, Olumuyiwa Oludayo, Colette Nsofor, Adeola Oyebode

**Affiliations:** Centre for Entrepreneurial Development Studies, Covenant University, Nigeria

## Abstract

This data article is an expression of data that reflects how students’ participation in the Hult Prize 2018 regional finals affects their decision to become entrepreneurs. The primary data was sourced using a questionnaire developed with Google doc form. Out of 120 students that participated in the Hult Prize 2018 regional finals in Nigeria, 103 of them responded. Their responses are as presented in this article. Such will be relevant to researchers who want to find out why students desire to become entrepreneurs and the best approach and timing to enable them.

## Specifications Table

**Table:** 

Subject area	*Economics and Business*
More specific subject area	*Entrepreneurship*
Type of data	*Tables, charts and figures*
How data was acquired	*The data is a primary data, which was sourced using a Google Doc form questionnaire.*
Data format	*Raw*
Experimental factors	*NA*
Experimental features	*NA*
Data source location	*Higher Education Institutions in Cameroun, Ghana, Nigeria, Uganda and United Kingdom, which participated in the Hult Prize 2018 Regional Finals in Nigeria.*
Data accessibility	*Data are available with this article.*
Related research article	*None.*

## Value of the data

• The data provides insight into the impact entrepreneurship programmes and competitions can have on students’ decisions to become entrepreneurs.
• With the data, researchers can explore the reasons why students want to become entrepreneurs.
• The data will also be valuable to researchers who want to find out the most suitable time to help students develop their entrepreneurial skills, based on their desire, and the kinds of responsibilities and experiences to expose them to.
• The data is relevant in revealing the areas students would likely launch their enterprises if given the chance to.
• The fact that the data spans across countries and disciplines suggests that inferences from studies that engage the data can be relevant across countries and disciplines.

## 1 Data

The data in this article is a primary data gathered on March 16, 2018 at the Hult Prize 2018 Regional Finals held in Covenant University, Nigeria. Out of 120 students that participated in the competition, 103 students responded. The data encapsulates the respondents’ reasons for participating in the programme, the role they played and how such affects their desire for entrepreneurship, when they would like to commence their entrepreneurial journeys and what areas they would likely venture into.

## 2 Experimental design, materials, and methods

A Google Doc questionnaire form was created specifically to harvest the data and used to collect the data. Out of 120 students that participated in the competition, 103 students responded. Their responses are as presented (Table 1, Table 2, Table 3, Table 4, Table 5 and Fig. 1, Fig. 2, Fig. 3, Fig. 4, Fig. 5, Fig. 6, Fig. 7, Fig. 8, Fig. 9, Fig. 10).

**Table 1:** Reason for participating in the Hult Prize competition.

**s/n**	**Options**	**Number of selections**
1	To join other students enabling global change	64
2	The alignment with Sustainable Development Goals (SDG)	46
3	To compete for US$1 million	35
4	To solve social problems	80
5	To learn and build my capacity	55
6	The need for an extra-curricular activity	13
7	Other	1

**Table 2:** What role(s) do you play on your team in your Hult Prize participation?.

**s\n**	**Options**	**Number of selections**
1	Research	49
2	Scouting for sponsorship and/or partnership	27
3	Community engagement	19
4	Project implementation	36
5	Product development	44
6	Strategy and Innovation	46
7	Sales and Marketing	24
8	Media and publicity	20
9	Other	3

**Table 3:** Reasons for the choice to start a business as influenced by involvement in the Hult Prize.

**s\n**	**Options**	**Number of selections**
1	To make global impact	83
2	To build a viable venture	47
3	Availability of market	34
4	Potential room for growth and scalability	34
5	Uniqueness of the project work on before	23
6	Other	2

**Table 4:** Reasons why respondents want to become entrepreneurs.

**s\n**	**Options**	**Number of selections**
1	A means of survival	10
2	To make money	32
3	To impact lives	98
4	To be my own boss	30
5	Other	1

**Table 5:** Areas respondents would like to venture into in commencing their entrepreneurial journey.

**s\n**	**Options**	**Number of selections**
1	Agriculture	55
2	Education	37
3	Environment	27
4	Health	24
5	ICT	37
6	Food/Drinks	10
7	Finance	6
8	Fashion	10
9	Commerce (Trading etc.)	13
10	Entertainment	5
11	Sports	3
12	Community development	27
13	Tourism	3
14	Other	4

**Fig. 1:**
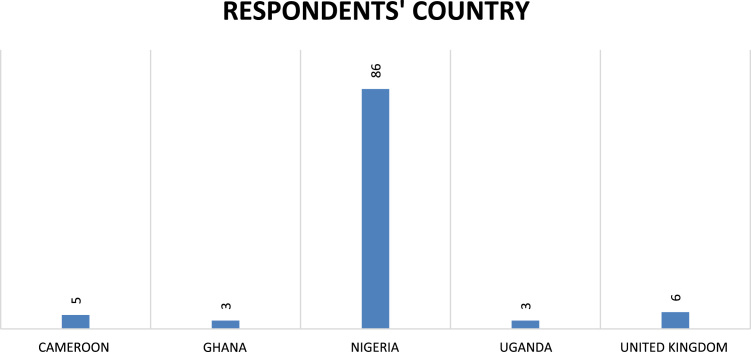
Country of respondents.

**Fig. 2:**
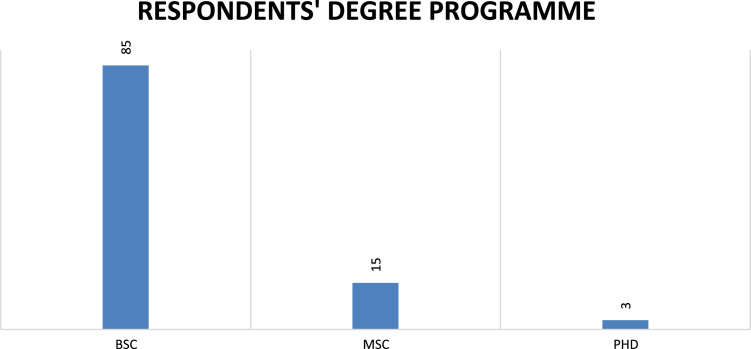
Degree programme of respondents.

**Fig. 3:**
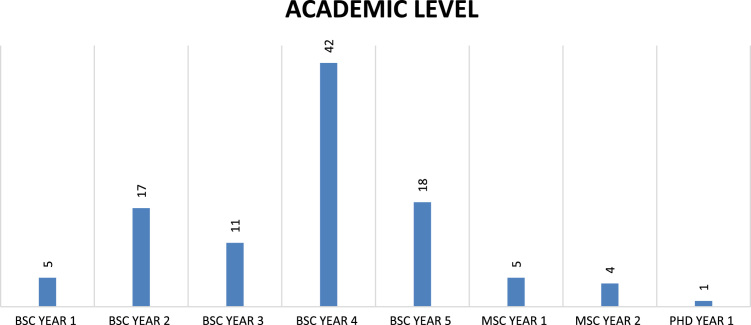
Academic level of respondents.

**Fig. 4:**
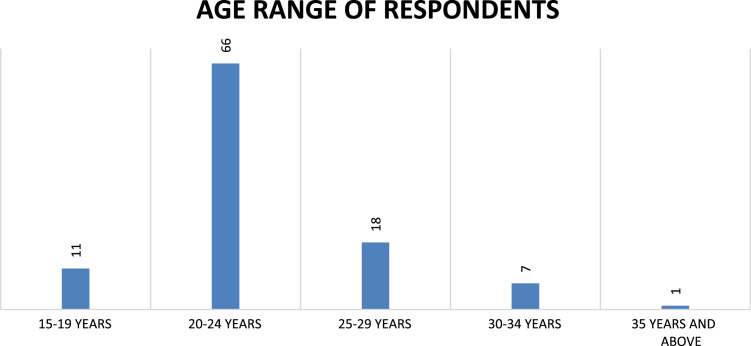
Age range of respondents.

**Fig. 5:**
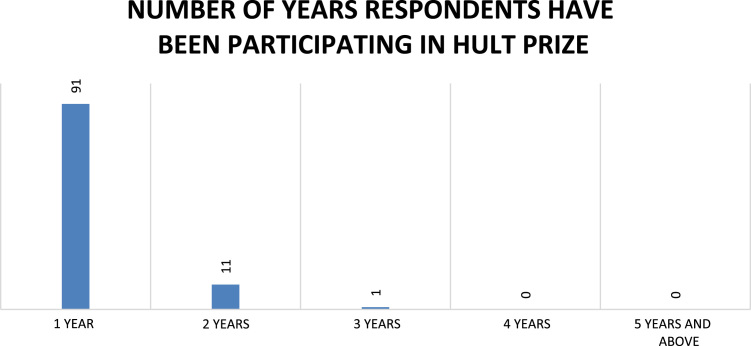
Duration of respondents’ participation in Hult Prize.

**Fig. 6:**
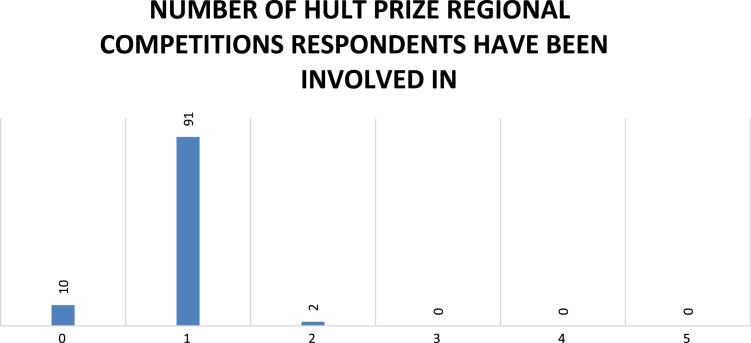
Number of Hult Prize regional competitions respondents participated in.

**Fig. 7:**
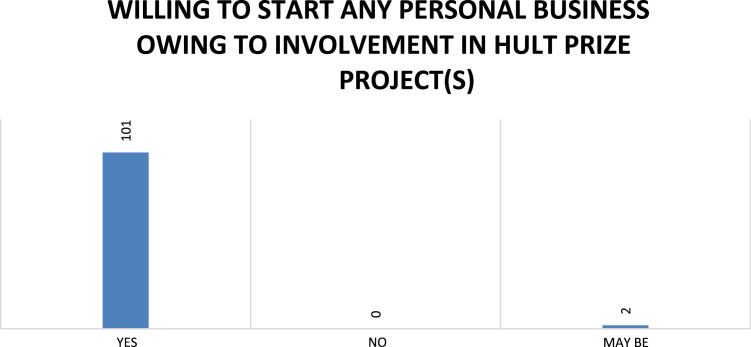
Respondents’ willingness to start a business owing to Hult Prize involvement.

**Fig. 8:**
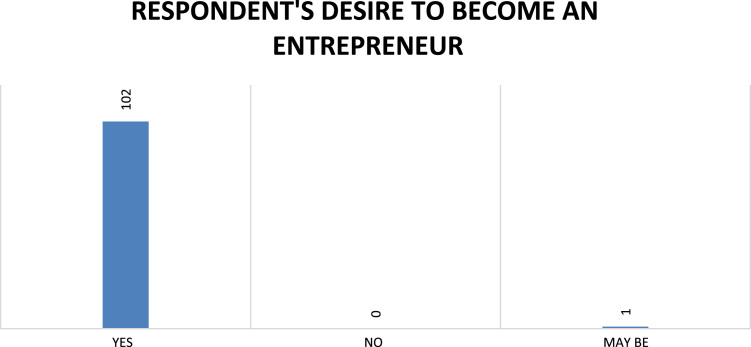
Respondents’ desire to become entrepreneurs.

**Fig. 9:**
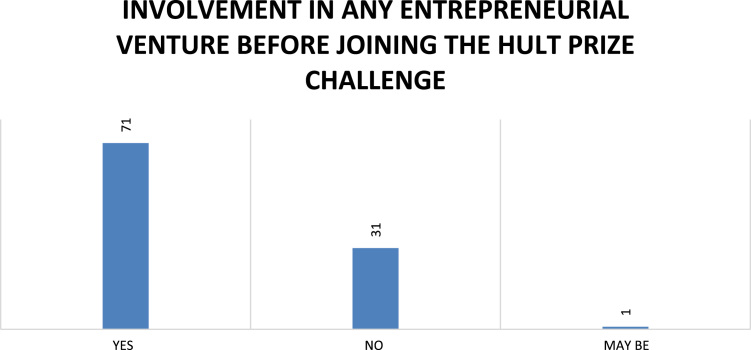
Respondents’ involvement in any venture before involvement in Hult Prize.

**Fig. 10:**
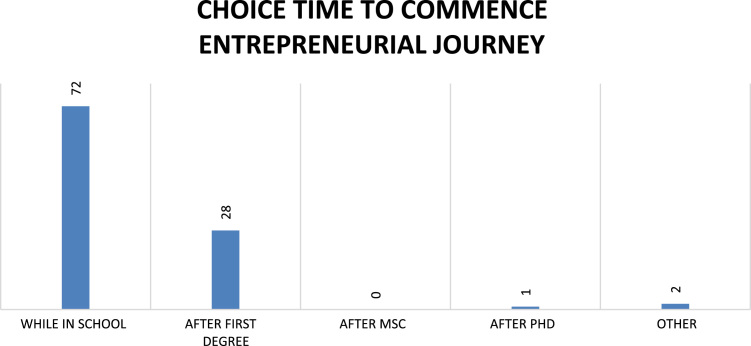
Respondents’ choice time to commence entrepreneurial journey.

